# Spatial coordination between chromosomes and cell division proteins in *Escherichia coli*

**DOI:** 10.3389/fmicb.2015.00306

**Published:** 2015-04-14

**Authors:** Jaan Männik, Matthew W. Bailey

**Affiliations:** ^1^Department of Physics and Astronomy, University of Tennessee, Knoxville, TN, USA; ^2^Department of Biochemistry and Molecular and Cellular Biology, University of Tennessee, Knoxville, TN, USA

**Keywords:** nucleoid, divisome, Z-ring, cell division, *Escherichia coli*, protocell, nucleoid occlusion

## Abstract

To successfully propagate, cells need to coordinate chromosomal replication and segregation with cell division to prevent formation of DNA-less cells and cells with damaged DNA. Here, we review molecular systems in *Escherichia coli* that are known to be involved in positioning the divisome and chromosome relative to each other. Interestingly, this well-studied micro-organism has several partially redundant mechanisms to achieve this task; none of which are essential. Some of these systems determine the localization of the divisome relative to chromosomes such as SlmA-dependent nucleoid occlusion, some localize the chromosome relative to the divisome such as DNA translocation by FtsK, and some are likely to act on both systems such as the Min system and newly described Ter linkage. Moreover, there is evidence that *E. coli* harbors other divisome-chromosome coordination systems in addition to those known. The review also discusses the minimal requirements of coordination between chromosomes and cell division proteins needed for cell viability. Arguments are presented that cells can propagate without any dedicated coordination between their chromosomes and cell division machinery at the expense of lowered fitness.

## Introduction

In most bacteria, the main macromolecular structure that is responsible for coordinating cell division with other cellular processes, including replication and segregation of chromosomes, is the Z-ring (Margolin, 2005; Adams and Errington, 2009; de Boer, 2010; Lutkenhaus et al., 2012). The Z-ring is organized by linear FtsZ-(proto)filaments that in *Escherichia coli* are anchored to the cell plasma membrane by FtsA and ZipA linker proteins. The assembly and disassembly of protofilaments can happen rapidly on the time-scale of seconds (Erickson et al., 2010). This dynamic nature of the Z-ring makes it susceptible to regulation by numerous protein factors that can tip the balance between the assembly and disassembly of filaments.

**FIGURE 1:**
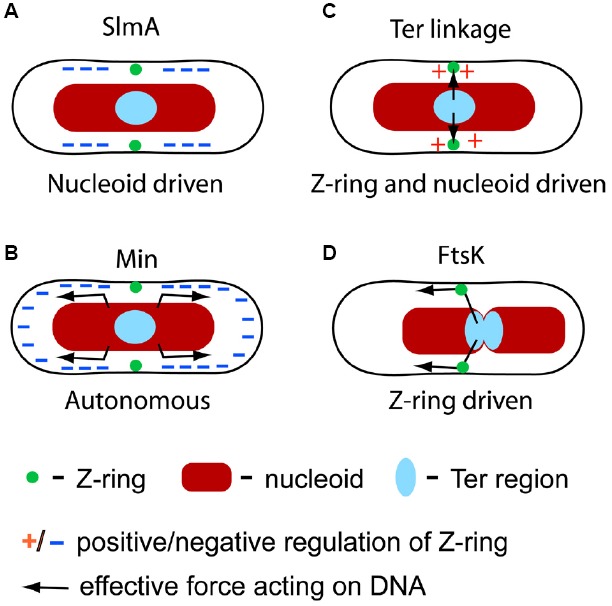
**Four molecular mechanisms that have been identified in spatial coordination between the chromosome and divisome in *E. coli.* (A)** SlmA-mediated nucleoid occlusion is a nucleoid driven mechanism which negatively regulates Z-ring formation in the vicinity of the chromosome except at the replication terminus region. **(B)** The Min system is independent of the nucleoid but it has been implicated in segregating and separating nucleoids. The Min system negatively regulates Z-ring formation at cell poles. **(C)** The Ter linkage is involved in determining the location of the Z-ring by a positive regulatory mechanism. The linkage is also involved in holding the Ter region fixed relative to the divisome. **(D)** FtsK translocase pumps DNA across the divisome in a directed manner leading to repositioning of chromosomes.

Formation of the Z-ring is the first step in bacterial cytokinesis. Once the Z-ring has formed it becomes a scaffold for over 30 other proteins that form a divisome complex (Liu et al., 2015). The divisome carries out septal envelope synthesis that leads to the pinching off of one daughter cell from the other. In wild-type *E. coli*, pinching off occurs very accurately in the middle of the mother cell between two separated daughter nucleoids (Trueba, 1982; Den Blaauwen et al., 1999; Männik et al., 2012). In mutant cells, the inaccurate placement of the Z-ring relative to nucleoids can lead to cells lacking chromosomal DNA completely, i.e., minicells (Adler et al., 1967) or to cells that have an incomplete set of genetic material, i.e., have guillotined nucleoids (Niki et al., 1991; Cook and Rothfield, 1999; Hendricks et al., 2000). *E. coli* cells have developed a number of molecular systems to prevent this outcome. These systems include nucleoid occlusion (NO), the Min system, the Ter linkage, and FtsK translocase (Figure 1). There is evidence that other mechanisms may also be involved. Here, we will review the aforementioned known molecular systems and discuss some hypothetical ones that have been implicated in spatially coordinating the divisome and chromosome. We will limit our discussion to *E. coli*; coordination systems in other bacterial species show divergent molecular origins (Adams et al., 2014; Monahan et al., 2014). We will expand our scope in the last part of this review where we will discuss the minimal requirements for the coordination between cell division proteins and chromosomes that are necessary for the survival of any cell.

## Nucleoid Occlusion

The early discussion of coordination between cell division and chromosome replication/segregation centered on the idea of NO (Hussain et al., 1987; Mulder and Woldringh, 1989; Woldringh et al., 1990; Wu and Errington, 2011). The idea of NO is based on observations that constrictions in dividing cells were excluded from the regions occupied by the nucleoids. Woldringh and others proposed that the inhibitory effect of the nucleoid is mediated by short range interactions stemming from the nucleoid which are related to transcription and translation (Woldringh et al., 1991). Zaritsky and Woldringh (2003) further refined the idea proposing that molecular crowding of the inner surface of the plasma membrane in the vicinity of the nucleoid was responsible for this inhibitory effect. These authors hypothesized that crowding results from the transertion process. In transertion, nascent membrane proteins are synthesized concurrently with yet ongoing transcription of their mRNA and are inserted into the cell plasma membrane while their translation occurs (Norris, 1995; Woldringh, 2002). Altogether, this process links DNA to the inner membrane by a molecular chain that includes RNA polymerase, nascent mRNA, ribosome, and nascent protein. The nascent protein is hypothesized to insert itself into the plasma membrane by its N-terminal domain while still being translated. Taking that approximately 1/3 of all proteins in *E. coli* are membrane proteins, the number of transertional linkages should be considerable at any given point of the cell cycle and could lead to significant membrane crowding (Woldringh, 2002). Zaritsky and Woldringh (2003) hypothesized that crowding could be lower in membrane areas adjacent to the replication terminus region because of the smaller density of highly expressed membrane targeted genes. This assumption suggests that Z-ring assembly is least inhibited in membrane regions adjacent to the replication terminus in accord with experimental observations.

The effect of transertion-related crowding on Z-ring formation was experimentally studied (Sun and Margolin, 2004). These authors observed that severing transertional linkages by blocking transcription with rifampicin treatment indeed allowed Z-rings to form over the nucleoids. Conversely, severing transertional linkages by blocking translation with chloramphenicol caused nucleoid compaction and exclusion of Z-rings from the membrane regions in the proximity of nucleoids. Furthermore, in SecA(ts) cells where protein insertion was thermally inactivated, the NO effect was still present even though the nucleoids did not segregate properly. The two latter observations therefore were not in accord with the transertion-based crowding ideas. To explain their data, the authors concluded that the nucleoid structure and packing density most likely plays a role in excluding the Z-rings from the regions adjacent to the nucleoid. Z-rings could localize “over” the expanded nucleoids but not over the compact ones. This idea has been supported by other measurements where nucleoid packing was altered (Sun and Margolin, 1998). So far, it remains unclear how the nucleoid packing density can influence Z-ring positioning especially during chloramphenicol treatment when the compacted nucleoid resides far from the cell membrane. Indirect effects of chloramphenicol and rifampicin treatment in these experiments could not be ruled out. In that light, more experiments are warranted to explore further the role of membrane crowding on Z-ring positioning.

## SlmA Mediated Nucleoid Occlusion

A major development in the understanding of NO came with the discovery of the SlmA protein from a synthetically lethal screen (Bernhardt and de Boer, 2005). Cells that lacked both *slmA* and *minCDE* were not able to divide in rich medium and gave rise to filamentous cells, indicating a defect in septation. The same authors also observed that some Z-rings could localize over unsegregated nucleoids in *slmA minCDE* cells suggesting SlmA played a role in Z-ring positioning. This role was also visible from studies of *slmA dnaA* cells in rich medium where a closing septum appeared in the middle of the nucleoid mass presumably because of the lack of a functional NO system (Bernhardt and de Boer, 2005). Spatial positioning of the Z-ring by SlmA is thought to result from a specific binding pattern of SlmA on *E. coli* chromosomal DNA. SlmA specific binding sites are distributed over the chromosome with a notable exception at the replication terminus region (Cho et al., 2011; Tonthat et al., 2011). Such a distribution, together with the inhibitory action of SlmA, can generate the NO effect, i.e., Z-rings do not assemble over chromosomal DNA except near the replication terminus region. Note that the replication terminus region, which positions itself at mid-cell in the later part of cell cycle, is in the vicinity of the membrane region where the Z-ring typically assembles (Figure 1A).

Two possible molecular mechanisms by which SlmA inhibits the formation of the Z-ring have been proposed. One model posits that SlmA causes depolymerization of FtsZ protofilaments (Cho et al., 2011; Cho and Bernhardt, 2013; Du and Lutkenhaus, 2014). *In vitro* assays show that depolymerization of FtsZ only occurs at a significant rate when SlmA is bound to DNA in its specific binding sites (Cho et al., 2011). Consequently, depolymerization of protofilaments takes place everywhere in the nucleoid except at the replication terminus region. Further characterization of the depolymerization process shows that it occurs in two steps (Du and Lutkenhaus, 2014). In the first step, DNA-bound SlmA attaches to the highly conserved C-terminal tail of FtsZ. In this binding, SlmA competes with other FtsZ regulators (MinC, ClpX) and interaction partners (ZipA, FtsA, ZapD). In the second step, further interactions occur that lead to protofilament breakage. The resulting breakage appears to minimally affect the GTPase activity of FtsZ (Cho et al., 2011).

In an alternative model, it was proposed that DNA-bound SlmA does not depolymerize protofilaments but instead captures them and renders them incapable of Z-ring formation (Tonthat et al., 2011, 2013). The authors showed that SlmA binds to its specific binding sites as a dimer of dimers. Once the initial nucleation has occurred, the dimers can cooperatively spread on DNA. Tonthat et al. (2011, 2013) proposed that these higher order SlmA structures capture FtsZ filaments. However, so far microscopy of FtsZ fusion proteins has not confirmed any co-localization between FtsZ protofilaments and extended SlmA structures within the cell.

While characterization of SlmA at the molecular level has been extensive, understanding its role and function at the cellular level is still limited. How could SlmA that is bound to chromosomal DNA inhibit Z-ring formation at the cell membrane? Models described in (Du and Lutkenhaus, 2014) and (Tonthat et al., 2013), although different in their interaction mechanism between SlmA and FtsZ, both assume that DNA-bound SlmA comes into proximity of the cell membrane to influence the localization of the Z-ring. However, DNA-bound SlmA within the nucleoid makes only limited contacts with the membrane and therefore would interact infrequently with membrane-bound FtsZ. Transertional linkages may help to facilitate these contacts (Tonthat et al., 2013) but the existing microscopy data indicates that SlmA is localized within the nucleoid rather than in the vicinity of the cell surface (Bernhardt and de Boer, 2005). Alternatively, Bernhardt and de Boer (2005) proposed that depolymerization of protofilaments can happen within the nucleoid. The latter mechanism would lead only to a weak site-selective effect of SlmA on Z-ring localization. Existing experimental data (Männik et al., 2012) indeed shows that SlmA does not convey a strong site-selective effect on Z-ring location in fast growth conditions. How effectively SlmA inhibits Z-ring formation in various growth conditions remains yet to be characterized.

Although SlmA mediated NO has received the most attention recently, there is strong evidence that additional mechanisms beyond SlmA can lead to a NO effect in *E. coli*. It was observed that cell division proteins are positioned in accordance with NO in cells lacking SlmA. The NO effect was distinctly present even when the shapes and sizes of these cells were strongly perturbed (Männik et al., 2012). It was also observed that a replication-inhibited and unsegregated nucleoid at mid-cell blocks Z-ring formation independent of the SlmA and SOS response (Cambridge et al., 2014). How these inhibitory effects of nucleoids are mediated at a molecular level is currently not known, but from these data it is clear that the SlmA-related mechanism is not the only one that realizes NO in *E. coli* cells.

## The Min System

The three Min proteins, MinC, MinD, and MinE, form a well-understood geometric positioning system for the Z-ring in *E. coli* that defines the cell’s geometric middle and prevents polar septations (Lutkenhaus, 2007, 2012; Shapiro et al., 2009; Moseley and Nurse, 2010). Fluorescent tagging of Min proteins has shown that MinC, MinD, and MinE exhibit remarkable oscillatory behavior in *E. coli* cells, moving back and forth between the two poles with a typical oscillatory period from 30 s to 1 min (Raskin and de Boer, 1999). Of these three proteins, only MinD and MinE are necessary to set up the oscillations while MinC, which follows and binds to MinD, acts as an inhibitor for Z-ring formation (Lutkenhaus, 2007). MinC binding to membrane-attached MinD activates its inhibitory function (Lutkenhaus, 2012). Due to the oscillations, the destabilizing effect of MinC on Z-ring formation is the strongest at the cell poles, where the time-averaged concentration of MinD-bound MinC is the highest. This negative regulation prevents minicelling at the poles. However, the Min system appears also to play a role in the precise localization of the Z-ring at midcell (Guberman et al., 2008).

In its inhibitory action, MinC resembles SlmA. MinC also binds to the conserved C-terminal domain of FtsZ and its subsequent interactions lead to breakage of the FtsZ polymer. GTPase activity of FtsZ is required for the MinC mediated breakage but at the same time MinC does not lead to increased GTPase activity of FtsZ (Dajkovic et al., 2008; Shen and Lutkenhaus, 2010). Measurements using *in vivo* reconstituted assays show how these two perhaps contradictory findings can be reconciled (Arumugam et al., 2014). Arumugam et al. (2014) propose that MinC dimers cap FtsZ filament ends. They also observe that FtsZ filaments lose monomers throughout its length. Such loss leads to breaks and gaps in the filament. In the absence of MinC these breaks could be annealed by the addition of new monomers but in the presence of MinC capping this would not occur and filaments become destabilized. The same authors also propose that MinC binding to FtsZ hinders protofilament bundling, which leads to further weakening of the Z-ring. Because of the several observed similarities between SlmA and MinC, it is tempting to speculate that a similar scenario also can be realized when SlmA depolymerizes FtsZ filaments.

Extensive modeling has been carried out to capture the oscillatory behavior of the Min system based on continuum models and stochastic simulations. For a cross-section of this work spanning from early to current models see (Meinhardt and de Boer, 2001; Kruse, 2002; Huang et al., 2003; Fange and Elf, 2006; Kerr et al., 2006; Halatek and Frey, 2012; Bonny et al., 2013). The oscillatory movement of MinD and MinE in the cell emerges in all of these models due to the ATP and MinE-modulated attachment of MinD to the plasma membrane. The computational models are able to semi-qualitatively reproduce experimentally measured oscillation patterns and oscillation periods. Although different models introduce slight variations in reactions occurring between MinD and MinE, they all can be categorized in mathematical terms as reaction-diffusion systems that exhibit Turing instability (Turing, 1952). Arguably the Min system in *E. coli* is the best studied example where the Turning instability mechanism leads to the formation of a dynamic pattern in a living organism.

The Min system functions autonomously from the nucleoid as shown convincingly in *in vitro* reconstituted assays (Loose et al., 2008; Ivanov and Mizuuchi, 2010; Schweizer et al., 2012). The same conclusion also can be drawn based on experiments with cells that lack nucleoids but have a functioning Min system. Remarkably, in these cells the Z-ring also can be placed relatively accurately in the middle of the cell (Sun and Margolin, 1998; Yu and Margolin, 1999; Pazos et al., 2014). Taking that the two daughter chromosomes separate from each other approximately at mid-cell in normal growth conditions, this system alone is perhaps sufficient to coordinate chromosomes and cell division proteins in *E. coli*. However, if the cell shape becomes aberrant (Männik et al., 2012) then the Min system and NO can define different locations for the cell division plane. In conflicting cases, it appears that the NO mechanism dominates over the Min system (Männik et al., 2012).

While the Min system is not directly involved in coordinating the Z-ring and chromosomes, an indirect involvement is possible (Figure 1B). Several authors have pointed out that deletion of the Min system leads to a small defect in chromosome segregation and in the separation of daughter nucleoids in *E. coli* (Mulder et al., 1990; Akerlund et al., 2002; Di Ventura et al., 2013; Jia et al., 2014). Di Ventura et al. (2013) have proposed that MinD, which is a homolog of ParA, binds to DNA. These authors propose that MinD oscillation and DNA binding provides a Brownian-ratchet mechanism for DNA segregation and separation (Di Ventura et al., 2013). Further experimental support to this interesting idea is still warranted.

## The Ter Linkage

The Min system and NO are negative regulators for cell division proteins, i.e., they inhibit Z-ring formation in certain locations of the cell. Recent research strongly suggests that there exists also a positive regulation mechanism in *E. coli*, which guides cell division proteins toward the replication terminus region of the chromosome (Bailey et al., 2014; Figure 1C). The presence of a positive regulation mechanism became evident in studies of cells that lacked both the Min system and SlmA-mediated NO. In these cells, the Z-ring positioned itself over the centers of segregating nucleoids instead of localizing at the cell poles or gaps between the nucleoids. The effect was particularly striking in cephalexin-treated cells that show many well separated nucleoids. Time-lapse measurements of *slmA min* cells showed that formation of the Z-ring commenced shortly after the arrival of the replication terminus region to the nucleoid center, even though transient associations of the Z-ring and the replication terminus region could be seen before the replication terminus centralized.

**FIGURE 2:**
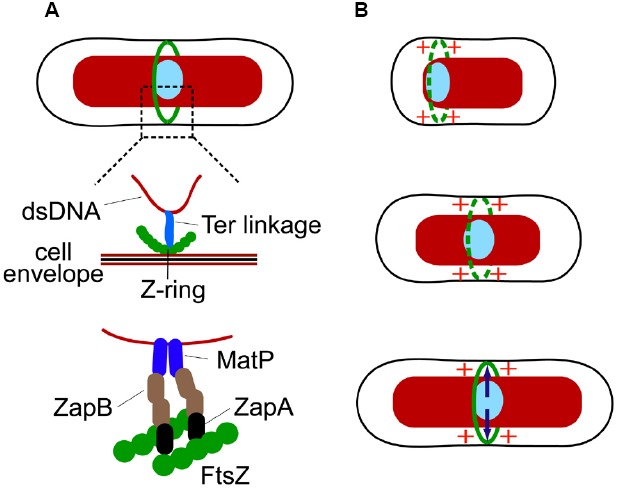
**The Ter linkage coordinates localization of both the Z-ring and the Ter macrodomain region of the chromosome. (A)** A putative connection between the DNA in the Ter region and the Z-ring via the Ter linkage. Schematics from top to bottom show progressive zoom-ins of the Ter region (light blue ellipse) and the Z-ring (green circles). ZapB in the linkage could form filaments or even filament networks. **(B)** Cell-cycle dependent processes involving the Ter linkage. Top: in the beginning of cell cycle the Ter macrodomain region is localized at the nucleoid periphery close to the new pole of the cell. Positive regulation from the Ter region promotes transient Z-rings to form in its vicinity. Middle: as the cell cycle progresses, the Ter region moves to the center of the nucleoid. It has been hypothesized that the motion is related to replication of the Ter region (Espeli et al., 2012). The positive signal from the Ter region continues to promote Z-ring formation in its vicinity. Bottom: as the Z-ring fully forms the Ter region becomes attached to the Z-ring through a link involving MatP, ZapB, and ZapA (indicated by arrows).

A different piece of evidence shows that the replication terminus region is anchored to the Z-ring (Espeli et al., 2012). The replication terminus region of *E. coli* chromosome forms a compact entity, termed the Ter macrodomain, which is organized by MatP proteins (Mercier et al., 2008; Dupaigne et al., 2012). MatP, like SlmA, is a DNA-binding protein. Interestingly, binding sites of these two proteins in *E. coli* chromosomal DNA are completely complementary—MatP specific binding sites, 23 total, can be found only in an 800 kb stretch around the *dif* sequence (located at 32.24′) in the replication terminus region while SlmA binding sites are located, essentially, everywhere else. Work by Espeli et al. (2012) has shown that the anchoring of the Ter region to the Z-ring occurs due to the MatP C-terminal interaction with the Z-ring associated protein ZapB (Espeli et al., 2012), which indirectly interacts with FtsZ through ZapA (Galli and Gerdes, 2010). The anchor, which we refer as the Ter linkage, connects the replication terminus region to the Z-ring through a chain of DNA-MatP-ZapB-ZapA-FtsZ. Only the nearest components in the Ter linkage are thought to interact (Figure 2A). The function of the linkage is not fully established but it appears to guarantee that the positioning of the chromosomes relative to the divisome does not change after the Z-ring has formed.

There are thus two sequential processes occurring that coordinate the Z-ring and the replication terminus region (Figure 2B). First, a signal from the replication terminus region promotes assembly of the Z-ring in its immediate vicinity. Second, after the Z-ring has formed the replication terminus region becomes linked to the Z-ring by a connection involving MatP, ZapB, and ZapA. Interestingly, ZapA and ZapB, and to lesser degree MatP, are also needed in the first step (Bailey et al., 2014). How exactly MatP, ZapB, and ZapA promote Z-ring formation in the vicinity of the Ter macrodomain is not known. It is possible that positive regulation itself is not a direct consequence of MatP, ZapA, and ZapB but results from some other molecular system that associates with the replication terminus region. MatP, ZapA, and ZapB may help to reinforce this signal by linking the source of the signal to the nascent Z-ring and strengthening its effect. At this point, the hypothesis has not been tested and the mechanistic details of the positive regulation are yet to be established.

## Effect of the Divisome on the Nucleoid—the FtsK Translocase

So far the discussion has focused on mechanisms where the nucleoid directly or indirectly determines the localization of cell division proteins. The opposite processes, in which the divisome affects positioning of the nucleoid, or at least part of it, are also present in *E. coli.* The Ter linkage, discussed in previous section, is one example where the divisome exerts its effect on the nucleoid. However, the Ter linkage appears to maintain rather than to actively re-arrange the chromosomal organization and positioning. Contrarily, DNA translocase FtsK (Begg et al., 1995) allows the divisome to actively re-arrange the *E. coli* chromosome in an ATP-dependent manner (Figure 1D). As is the case with the Ter linkage, the activity of FtsK seems to affect the Ter region of the chromosome (Deghorain et al., 2011; Stouf et al., 2013). Since the Ter region comprises about 20% of the total *E. coli* chromosome its re-arrangements have global implications to chromosome organization.

FtsK translocation activity leads to positioning of chromosomal *dif* site in the divisome during late stages of the cell cycle. The *dif* site is a 28 bp sequence in the replication terminus region where chromosome dimers are resolved by the XerCD recombinase. FtsK is capable of pumping DNA on both chromosome arms toward the *dif* site. The directionality in pumping is due to KOPS (FtsK orienting/polarizing sequence), which are oriented in opposite directions on the left and right arms of the *E. coli* chromosome. FtsK loads onto DNA at KOPS sites in a specific orientation. Unidirectional pumping by FtsK then leads to the movement of the *dif* site toward the divisome irrespective of which chromosome arm FtsK was loaded upon (Sherratt et al., 2010). Loading and translocation can only occur *in vivo* when FtsK is localized in the divisome where it forms hexameric units (Bisicchia et al., 2013). The translocation through the barrel in the FtsK hexameric assembly takes place at a rate of about 5 kb/s and stops when FtsK reaches the *dif* site (Sherratt et al., 2010). FtsK translocation activity releases most DNA-binding proteins from the translocated region of the chromosome (Lee et al., 2014). Among others, MatP proteins are also released. The latter leads to dissociation of the Ter linkage at late stages of cytokinesis (Stouf et al., 2013). It is likely that the Ter linkage facilities the activity of FtsK by maintaining this chromosomal region physically in the proximity of the divisome.

FtsK activity on DNA is not limited to translocation. At *dif* sites, FtsK is responsible for activation of the XerCD system that resolves chromosome dimers. FtsK has also been implicated in activating Topo IV (parC), which removes chromosome catenates (Espeli et al., 2003).

## Minimal Requirements for Coordination

**FIGURE 3:**
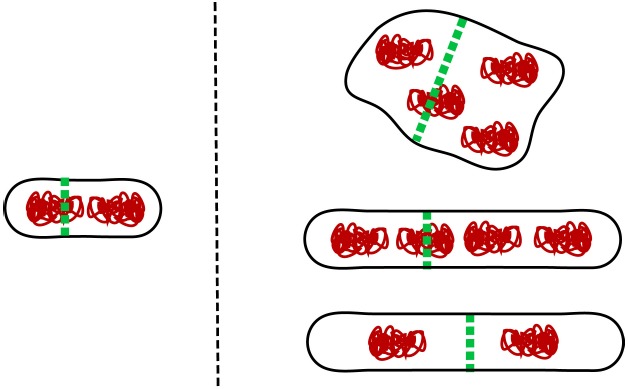
**Larger cell size mitigates lack of spatial coordination between cell division machinery and genetic information.** A small cell (left) requires a sophisticated apparatus to coordinate cell division and chromosomes. For larger cells (right) the requirements for coordination are more relaxed. This applies in particular for multi-nucleoid cells where despite some genetic material being damaged during division the majority of daughter cells still receive a full complement of genes and remain viable. At least in rod shapes, long diploid cells can also give rise to viable population even when their division plane placement is random.

Interestingly, none of the four molecular systems that have been discussed in this review are strictly essential in *E. coli*. One can delete *slmA* and *minCDE* together with either *matP*, *zapA*, or *zapB* from *E. coli* but the cells still remain viable in slow growth conditions (Bailey et al., 2014). Moreover, even though FtsK is essential, its DNA translocating domain (C-terminal domain) is not (Sherratt et al., 2010). Is it possible that *E. coli* harbors some additional molecular system that coordinates its divisome and chromosome, and this yet to be discovered system is indispensable? As discussed above, there is strong evidence that the NO effect can occur without SlmA. Although the molecular bases of this mechanism remains unknown it could be an essential mechanism. Alternatively, it is possible that there are no indispensable mechanisms that coordinate cell division and chromosome segregation in *E. coli*. One can ask what minimal coordination is needed between cell division proteins and chromosomes for any cell (not only *E. coli*) to propagate. For propagation of a sizeable cell population, the ultimate limit appears to be that on average more than half of the nucleoids need to survive cell division undamaged and emerge in newborn daughter cells (note that small cell populations can go extinct even when more than half of the nucleoids survive cell division). One way to fulfill this requirement in cells that lack any dedicated coordination mechanism between cell division and chromosomes is to increase cell size. If the division plane is placed randomly in the cell then the probability to produce viable daughter cells increases as the cell size increases (Figure 3). Multi-nucleoid cells are more likely to produce two viable daughters upon random placement of the division plane at the expense of losing some genetic material. However, rod-shaped bacterial cells with two nucleoids can also give rise to a viable population when their sizes are sufficiently large. In the latter case, it is assumed that the physical size of the nucleoid does not depend on cell size and nucleoids are randomly placed in the cell before division. It remains to be proven if viable populations also can emerge in other cell geometries under these assumptions. Interestingly, as the systems depicted in Figure 1 are progressively deleted from *E. coli*, the cells become larger (longer) but remain viable in slow growth conditions (Bailey et al., 2014) in accordance with this hypothesis.

Although large cells can cope with random placement of division planes, their fitness is very low because these cells lose a substantial amount of resources when they produce unviable cells or guillotine nucleoids. Mechanisms that coordinate cell division proteins and chromosomes are thus essential for cellular fitness. These mechanisms are highly efficient in modern bacteria. The probability that wild type *E. coli* produces minicells has been estimated to be less than 0.03% (Niki et al., 1991). It is, however, plausible that early protocells did not have any dedicated coordination systems. This argument is supported by findings that different bacterial species have evolved very different molecular mechanisms that coordinate divisomes and chromosomes (Monahan et al., 2014). As a corollary to this discussion, the lack of these coordination systems would imply that early protocells were perhaps larger than present day bacteria. Alternatively, early protocells might have had mechanisms that provided some coordination between division planes and chromosomes but which were not specifically dedicated for the task. For example, in a rod-shaped bacterial cell, membrane mechanics dictates that divisions which partition a mother cell into two equal halves are energetically more favorable than asymmetric divisions (Shlomovitz and Gov, 2009). Also, it is likely that chromosomes could be pushed mechanically away as the division septum closes preventing them from being guillotined or that chromosomes could provide enough mechanical hindrance to prevent the septum from closing in cells that lack strong cell wall. These mechanisms could still be present in modern *E. coli* even though their influence is overridden by the more efficient molecular systems such as Min, SlmA, Ter linkage, and FtsK.

## Concluding Remarks

As this review emphasizes, there are several modular pathways coordinating chromosomal positioning with cell division in *E. coli*, which are redundant, at least, in slow growth conditions. Moreover, there is evidence that, in addition to the mechanisms known so far, there are other types of coordination which have not been described yet. All the mechanisms lead to increased cellular fitness but are not essential for cell viability. Perhaps surprisingly, one of the conclusions of this review is that the *E. coli* cell can cope with very limited coordination between cell division proteins and chromosomes.

### Conflict of Interest Statement

The authors declare that the research was conducted in the absence of any commercial or financial relationships that could be construed as a potential conflict of interest.
